# On-Chip Glass Microspherical Shell Whispering Gallery Mode Resonators

**DOI:** 10.1038/s41598-017-14049-w

**Published:** 2017-11-02

**Authors:** Chenchen Zhang, Alexander Cocking, Eugene Freeman, Zhiwen Liu, Srinivas Tadigadapa

**Affiliations:** School of Electrical Engineering and Computer Science, Materials Research Institute, The Pennsylvania State University, University Park, Pennsylvania, 16802 USA

## Abstract

Arrays of on-chip spherical glass shells of hundreds of micrometers in diameter with ultra-smooth surfaces and sub-micrometer wall thicknesses have been fabricated and have been shown to sustain optical resonance modes with high *Q-*factors of greater than 50 million. The resonators exhibit temperature sensitivity of −1.8 GHz K^−1^ and can be configured as ultra-high sensitivity thermal sensors for a broad range of applications. By virtue of the geometry’s strong light-matter interaction, the inner surface provides an excellent on-chip sensing platform that truly opens up the possibility for reproducible, chip scale, ultra-high sensitivity microfluidic sensor arrays. As a proof of concept we demonstrate the sensitivity of the resonance frequency as water is filled inside the microspherical shell and is allowed to evaporate. By COMSOL modeling, the dependence of this interaction on glass shell thickness is elucidated and the experimentally measured sensitivities for two different shell thicknesses are explained.

## Introduction

Whispering gallery mode (WGM) resonances in optical cavities have been studied for more than a century since the interaction of electromagnetic waves with dielectric spheres was first observed in late 1900’s^1,2^. Since the first experimental observation in 1960’s^3^, the WGM optical resonances have been demonstrated to be supported within several structures with an axis of rotational symmetry such as microdroplets^4,5^, microtubes^6–8^, microbottles^9,10^, microspheres^11–13^, microrings^14,15^, microdiscs^16,17^, microbubbles^18,19^, and microtoroids^20^. WGM relies upon total internal reflection at the cavity interface. To induce a resonance mode, an adiabatically tapered fiber is placed in close proximity to the resonator structure to evanescently couple the light. A large refractive index contrast between the cavity and the surrounding medium strongly confines the WGMs resulting in resonances with very high *Q-*factors of 10^7^–10^9^ ^21,22^. Conversely, a low refractive index contrast facilitates extension of the modal profile beyond the confines of the resonator medium allowing for the optical radiation to interact with the surrounding medium and thus enabling sensor designs with exceptionally high sensitivity – albeit at the expense of the *Q*-factor. In general, changes in either the cavity geometry or the refractive index contrast between the cavity and surrounding medium perturb the resonance characteristics of the confined optical modes and can be used for sensing applications. The extreme level of sensitivity afforded by WGM resonators has elicited intense research in realizing sensors based on these structures^23^. To date, two kinds of WGM optical resonator configurations have been explored: (i) microsphere, microbottle, and microbubble structures formed by individually melting or machining and polishing fibers and capillaries of suitable dielectric materials to form highly smooth and axisymmetric structures; and (ii) on-chip microfabricated microring, microdisk, and microtoroid structures from suitable dielectric materials. Unlike solid structures such as spheres and discs, hollow structures such as cylindrical and spherical shells have two surfaces and offer the advantage of coupling the light through the outer surface whereas the inner surface can be engineered to induce perturbations for sensing. Microtube, and microbottle based sensors have been reported in a configuration commonly known as optofluidic ring-resonator (OFRR) sensors^8,24,25^ where the analyte fluid interacts with the optical resonance through the inner surface of the shell. However, until now all OFRR sensors have been fabricated by glass blowing techniques from individual capillaries where the physical characteristics of these structures are not easily controlled or reproducible. On the other hand, on-chip microring, microdisc and microtoroid based sensors are able to leverage the reproducibility afforded by microfabrication techniques and the economy of wafer scale manufacturing methods. However, in these resonators it is much harder to achieve a clean interface with fluidic analyte medium since in most typical configurations both the resonators and the tapered fiber are exposed to these fluids. Recently, chip-scale glass blowing techniques have been demonstrated to create hemispherical and toroidal structures from borosilicate glass and fused silica^26,27^. These structures consist of glass microspherical shells with radii ranging from 0.1 mm to >1 mm and are proposed here for use as WGM resonator structures. What is really significant is that these structures are highly reproducible and can be integrated with on chip microfluidics to achieve high performance WGM OFRR structures for sensing applications. Furthermore, the thickness of the microspherical shell structures on the chip can be precisely tailored to achieve optimal interaction with the fluid within while maintaining very high *Q-*factor for the optical resonance. These glass microspherical shell structures are also ideally suited for cavity optomechanical applications for sensitive detection of mechanical motion and quantum optomechanical experiments^28^ which can be further enhanced by operating these systems at exceptional points^29,30^. Hence, the microspherical shell structures can be utilized for a multitude of optical resonance based sensing applications including motion, temperature, pressure, (bio)chemicals etc.

**Figure 1:**
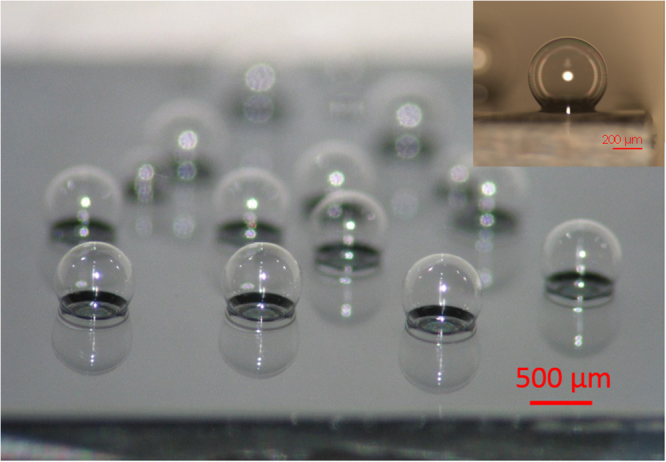
Chip-scale glass microspherical shells blown on silicon substrate. Inset shows a near perfect glass microspherical shell with a sphericity of 0.996.

In this paper, we demonstrate the first chip-scale, borosilicate glass microspherical shell, optical resonators with high-*Q* factors fabricated by glassblowing techniques and demonstrate the potential of these structures for on-chip sensing applications. We present a model for microspherical shells with diameters ranging from 230 µm to 1.2 mm and shell thicknesses of 300 nm to 10 μm. Figure 1 shows an array of the fabricated on-chip, glass microspherical shells with the equatorial planes above substrate. The on-chip integration of highly symmetric and smooth surface, closed spherical shell structures, can allow for the realization of WGM based in-line microfluidic (bio)chemical sensors where the analyte fluid interacts with the optical resonance through the inner surface of the shell. Here we demonstrate and model the thermal sensing capability of the glass microspherical shell resonator. Furthermore, we show a proof-of-concept liquid core sensor by sensing the index of refraction change from water to air and confirm the phenomenon with a model.

## Methods

**Figure 2:**
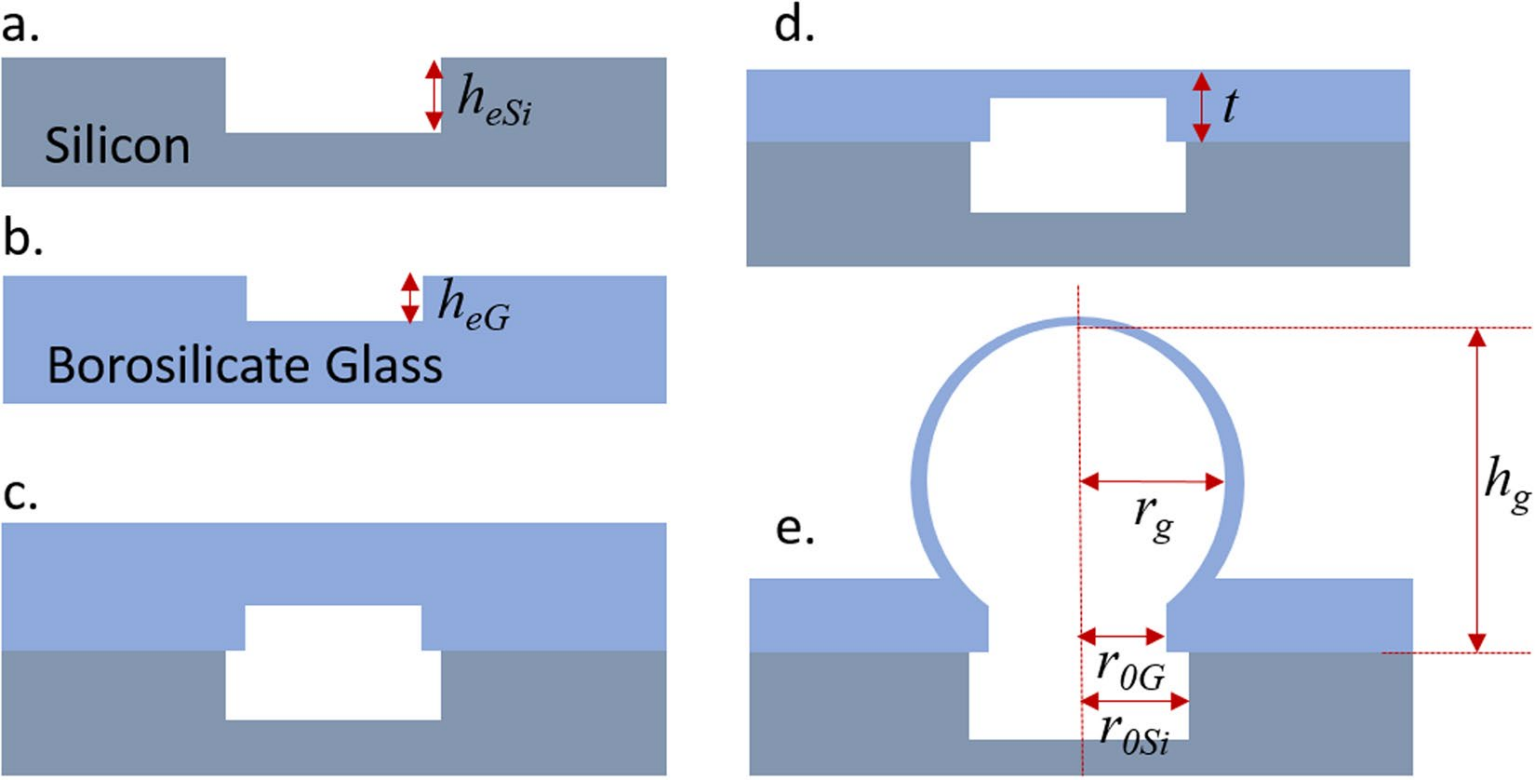
(**a**) Silicon wafer is patterned and plasma etched to a depth of 250 µm to define circular pits. (**b**) Borosilicate glass wafer is optionally patterned and plasma etched to define *h*_*eG*_ µm deep circular features. (**c**) The two wafers are aligned and anodically bonded. (**d**) Borosilicate wafer is thinned down to a thickness of *t* µm in hydrofluoric acid. (**e**) Glass microbubble is blown at 775 °C in a vacuum oven maintained at a pressure of 100 Torr.

The glass microbubbles were fabricated on 500 µm thick silicon substrate. First, circular features were patterned using positive photoresist and the silicon was etched to a depth of *h*_*eSi*_ = 250 µm using deep silicon etching process to realize cylindrical cavities as schematically shown in Fig. 2(a). Second, Corning^®^ 7740 borosilicate glass wafer was optionally patterned with smaller circles than on silicon using positive photoresist and 4 µm of nickel was electroplated as an etch mask. After removal of the photoresist in acetone, the borosilicate wafer was etched to a depth of *h*_*eG*_ µm using a modified ICP-RIE high-aspect ratio glass etch process^31^. Thereafter, the nickel, chrome and gold layers were stripped from the borosilicate wafer using wet etchants resulting in a cross-sectional profile as shown in Fig. 2(b). The etched silicon and optionally etched borosilicate glass wafers were aligned to result in concentric circles and anodically bonded at a pressure of 1.35 atmosphere (1026 Torr) at 400 °C to form the bonded cavity as shown in Fig. 2(c). The bonded wafer was diced into chips and the borosilicate layer was thinned down to a total thickness of *t* µm from the un-etched side in 49% hydrofluoric acid as shown in Fig. 2(d). The bonded chip was thereafter heated on a silicon nitride ceramic heater to a temperature of 775 °C in a vacuum oven maintained at 0.13 atmosphere (100 Torr) for 45 seconds and was rapidly cooled down to ambient temperature. At 775 °C, the borosilicate glass softens and begins to expand outwards into a spherical shell in response to the high pressure created within the sealed cavity at this elevated temperature and the external vacuum pressure^26^. The blown glass microspherical shell is schematically illustrated in Fig. 2(e). While the dicing step can be performed after the glass blowing step and the entire process can be done at wafer level, in this work we fabricated the glass microbubbles at chip scale due to the small sized heater used in this work. However, if the bubbles are blown at wafer scale, extreme care must be exercised during the dicing step to prevent contamination of the microspherical shell surface with particulate or other dicing related debris and residues.

## Results and Discussion

### Chip Scale Microspherical Shells

Referring to Fig. 2, the final height, *h*_*g*_, that the sphere develops is a function of the heater temperature *T*_*f*_ (in Kelvin), the pressure in the vacuum oven *P*_*f*_, the pressure *P*_*s*_ and temperature *T*_*s*_ (in Kelvin) at which the cavity is sealed, the etched depth *h*_*eSi*_ and *h*_*eG*_, and the radius *r*_*0Si*_ and *r*_*0G*_ of the etched cavity in the silicon and glass wafers respectively and is given by^26^:

1 $${h}_{g}=\frac{{[(3{V}_{g}+\sqrt{{r}_{0Si}^{6}{\pi }^{2}+9{V}_{g}^{2}}){\pi }^{2}]}^{2/3}-{r}_{0Si}^{2}{\pi }^{2}}{\pi {[(3{V}_{g}+\sqrt{{r}_{0Si}^{6}{\pi }^{2}+9{V}_{g}^{2}}){\pi }^{2}]}^{1/3}}$$

where

2 $${V}_{g}=(\frac{{P}_{s}{T}_{f}}{{P}_{f}{T}_{s}}({h}_{eSi}\pi {r}_{0Si}^{2}+{h}_{eG}\pi {r}_{0G}^{2})-{h}_{eSi}\pi {r}_{0Si}^{2})$$

The radius of the glass microsphere *r*_*g*_ can now be calculated as

3 $${r}_{g}=(\frac{{h}_{g}^{2}+{r}_{0Si}^{2}}{2{h}_{g}})$$

The sphericity of the blown glass microspherical shells Ψ is defined as^32^:

4 $${\rm{\Psi }}=(\frac{{\pi }^{\frac{1}{3}}{(6{V}_{g}^{\text{'}})}^{\frac{2}{3}}}{{A}_{g}})$$

where the *V*_*g*_′ and *A*_*g*_ are the effective volume and surface area respectively of the glass microspherical shell region above the top-surface of glass substrate and are expressed in terms of (*h*_*g*_|_*exp*_ − *t*), *r*_*g*_|_*exp*_, and *t* in eqs (5) and (6) as

5 $${V}_{g}^{\text{'}}=\frac{\pi }{3}{({{h}_{g}|}_{\exp }-t)}^{2}(3{{r}_{g}|}_{\exp }-({{h}_{g}|}_{\exp }-t))$$

6 $${A}_{g}=\pi ({{h}_{g}|}_{\exp }-t)(4{{r}_{g}|}_{\exp }-({{h}_{g}|}_{\exp }-t))$$

Table 1 lists the calculated *h*_*g*_ and radius *r*_*g*_ and experimentally measured values of *h*_*g*_|_exp_ and radius *r*_*g*_|_exp_ of various glass microspherical shells. A fairly good agreement between the calculated and experimental values of the heights and radii of the blown glass microspherical shells is found with a maximum error of <~20%. The calculated sizes of the microspherical shells are a sensitive function of the temperature and pressure at which these structures are sealed and blown. The experimental pressure and temperature are measured as global parameters at the system level of wafer bonder, heater, and vacuum oven pressure. Uncertainties in the actual temperatures and pressures at the individual microspherical shell level versus the global parameter values used in the calculations are considered to be the main reason for the observed discrepancy between the calculated and observed dimensions of the microbubbles. However, it must be emphasized that the process is highly reproducible across a single chip. For example, on a 1 cm × 1 cm chip on which more than 20 microspherical shells were blown simultaneously, the measured diameters of eight microscope-viewable shells was determined to be 1001.84 μm ±5.74 μm; i.e., a dimensional variation of ~0.5%.

**Table 1:** Calculated and experimentally measured values of the glass microspherical shell dimensions for the given glass blowing conditions. For devices where glass wafer is not etched prior to bonding, *r*_*0G*_ and *h*_*eG*_ are not applicable.

*Bubble*	*r*_*0Si*_ (μm)	*r*_*0G*_ (μm)	*h*_*eG*_ *(µm)*	*t (µm)*	*T*_*f*_ (K)	Theory		Experiment			Wall Thickness (*µm*)	Images of blown glass microbubble^†^
						*h*_g_*	*2r*_g_*	*h*_*g*_ *|*_*exp*_ *	*2r*_*g*_ *|*_exp_*	Ψ		
1	250	NA	NA	100	1023	1055	1115	1041	1197	0.9640	6.7	
2	100	NA	NA	100	1048	592	609	651	744	0.9511	8.4	
3	75	NA	NA	100	1048	491	503	526	614	0.9319	8.6	
4	100	NA	NA	50	1048	592	609	610	636	0.9875	2.2	
5	75	NA	NA	50	1048	491	503	568	554	0.9960	1.4	
6	40	NA	NA	50	1048	326	331	361	345	0.9915	1.1	
7	150	90	55	85	1048	792	820	729	713	0.9915	0.3	
8	75	65	55	85	993	510	520	466	404	NA	1.0	
9	40	35	55	85	993	278	281	326	231	0.8286	NA	

*h_*g*_, *h*_*g*_ *|*_*exp*_ *, r*_*g*_ and *r*_*g*_ *|*_*exp*_ are given in μm; *P*_*f*_ = 13 kPa, *T*_*s*_ = 673 K, *P*_*s*_ = 135 kPa, *h*_*eSi*_ = 250 µm; ^†^ **–** red scale bar in the images represents 250 µm.

The position of the equatorial plane of the glass microspherical shell is critical to obtaining WGM optical resonance. The optical modes are localized on the equatorial plane and are sustained only when the equatorial plane is above the substrate with minimal coupling loss to the substrate. In our initial experiments, the bonded silicon-glass substrates with sealed cavities were heated at ambient atmospheric pressure to blow the glass bubbles and resulted in hemispherically shaped shells. In these devices no optical resonance was obtained due to significant loss into the substrate. This situation was remedied by changing the glass blowing step to a vacuum ambient rather than at atmospheric pressure. The vacuum ambient during the glass blowing step raises the pressure difference relative to the sealed cavity pressure and enhances the expansion of microspherical shell volume to develop into near spherical structures with the equatorial plane located above the substrate for all shell sizes as shown in the last column of Table 1. The sphericity of the blown glass microspherical shells quantifies the relative height of the equatorial plane with respect to the glass substrate regardless of shell sizes. Sphericities in the range of 0.985–0.996 were measured for the shells #4–#7 which indicates that near-spherical glass shells were achieved in this work. Smaller sphericities were observed in glass microspherical shells #1–#3 blown out of thicker glass substrates. The excess material in these thicker glass substrates was observed to result in a lateral expansion at the shell-base during the glass reflow process. This visible lateral expansion at microspherical shell-base could be eliminated by reducing the thickness of the bonded glass layer which resulted in near spherical bubbles. Following optical resonance measurements, microspherical shells were cleaved at the equatorial plane and the sidewall thicknesses were measured using a scanning electron microscope (SEM). For glass microspherical shells blown from 100 µm thick glass layer, #1–#3, the thickness of the shell wall thickness ranged from 6.7 µm–8.6 µm whereas reducing the thickness of the glass substrate to 50 µm, shells # 4–#6, resulted in a wall thickness of 1.1 µm–2.2 µm. Plasma etching of the glass substrate in microspherical shells #7–#9 followed by the subsequent thinning of the glass substrates to realize even thinner glass regions of 30 µm resulted in either spherical or vertically elongated shells depending upon the radius and the enclosed cavity volume. Based on the volumetric redistribution of the glass covering the cavity opening into the spherical shell, the shell wall thickness can be estimated and agrees well with the measured thicknesses for all microspherical shells. For the etched glass substrates with a substrate glass thickness of 30 µm, shells with wall thicknesses as small as 300 nm were obtained. Furthermore, if a microspherical shell was overblown and was split open on the top, e.g. the broken shell seen in the background in the image of shell #7, optical resonance could still be sustained, so long as the remaining structure presented a spherical profile at the equatorial plane. Thus, through accurate control of: (i) the etched cavity geometries and dimensions, (ii) glass substrate thickness via micromachining, and (iii) the sealing and blowing conditions, wafer level glass blowing process can be customized to achieve glass microspherical shells of various sizes, sphericities, and wall thicknesses. The ultra-smooth surfaces obtained through the glass reflowing process are ideally suited for sustaining ultrahigh-*Q* optical resonances.

### Characterization of Optical Resonance in Microspherical Shells

**Figure 3:**
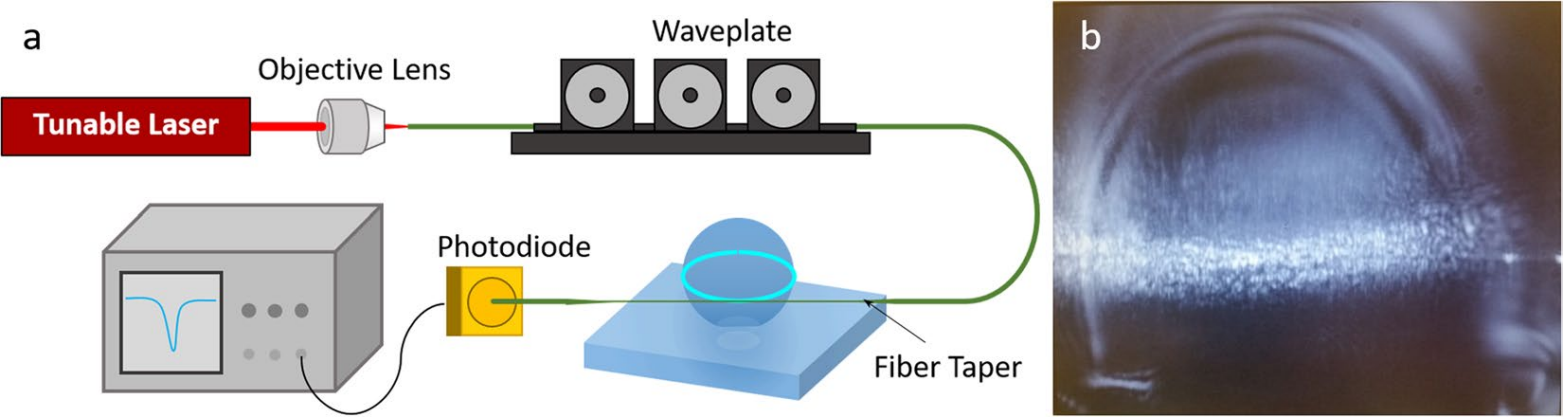
(**a**) Schematic illustration of the experimental set-up for the measurement for the WGM resonance in  microspherical shells. (**b**) Optical image showing the light confined to the equatorial plane of microspherical shell #9 upon evanescent coupling of the light through the tapered fiber.

The experimental set-up used for characterizing optical resonance in the glass microspherical shells is shown in Fig. 3(a). The excitation source consists of a tunable 760 nm laser (Thorlabs, TLK-L780M). The laser tuning was driven via a triangle wave at 10 Hz and corresponds to 15 GHz (Δλ = 28.87 pm) shift from the center wavelength of 760 nm. The light was evanescently coupled to the resonator via a tapered optical fiber. The fiber was fabricated using a hydrogen torch placed in the middle of the fiber and then being pulled at a constant rate from both ends. The polarization of the incident laser was adjusted using a fiber polarization controller to optimize coupling efficiency. After passing by the resonator and the fiber taper, the transmitted light was monitored using a photodiode (Thorlabs DET36A). Excitation of the resonance modes sustained in the equatorial plane of the glass microspherical shells manifest as dips in the transmission spectrum. The full width at half maximum (FWHM) of the transmission dips indicates the quality  factor of the resonance. Figure 3(b) shows an optical micrograph of microspherical shell #9 with a mode in which the light is confined to the equatorial plane of the  microspherical shell.

Optical resonance modes in WGM resonators occur when the coupled light can constructively interfere with itself by completing integral number of cycles for each revolution around the shell’s equatorial circle. Assuming that the mode is tightly confined within the resonator medium, for a laser wavelength of *λ*, the condition for WGM resonance in a dielectric annulus of radius *r* can be expressed as 2πn_*eff*_ *r* = *m*λ, where *n*_*eff*_ is the mode index; and *m* is the azimuthal mode number and corresponds to integral number of orbital wavelengths^33^. Table 2 lists the physical dimensions of the various microspherical shells studied in this work, the corresponding experimentally measured highest *Q-*factor, and the various resonance parameters calculated through COMSOL^®^ finite element simulation of the optical resonance characteristics. Azimuthal mode number *m* is calculated from the equation of 2π*n*_*eff*_ *r* = *m*λ with λ = 760 nm and *n*_*r*_ = 1.467 for borosilicate glass is used to estimate *n* *eff*. Eigen-frequencies *f*_*nml*_ were simulated with azimuthal mode number *m*, refractive index of bulk borosilicate glass *n*_*r*_ and shell geometry. Effective refractive index *n*_*eff*_ can be expressed as *n*_*eff*_  = *m*c/(2π*r f*_*nml*_), where *f*_*nml*_ is the simulated resonance frequency of fundamental TE mode, and c is the speed of light in vacuum. The effective refractive index is an indicator of how well the mode is confined within the glass shell and the thinner the shell thickness, the lower is its value as can be seen in Table 2.

**Table 2:** Optical characteristics of blown microbubbles.

*Bubble*	Experiment			COMSOL Simulation				
	Diameter (µm)	Wall Thickness (*µm*)	Highest *Q-*factor	Resonance Frequency *f*_*nml*_ *(THz)*	Azimuthal mode number *m*	Free Spectral Range	Finesse in 10^3^	Effective Refractive Index *n*_*eff*_
1	1197 ± 5	6.7	8.09 × 10^6^	$396.40\mp 0.02$	7259 ± 30	102 pm (54.4 GHz)	1.10	1.46091 ± 0.00002
2	744 ± 5	8.4	4.34 × 10^6^	$397.12\mp 0.04$	4512 ± 30	164 pm (87.5 GHz)	0.95	1.45831 ± 0.00004
3	614 ± 5	8.6	4.02 × 10^6^	$397.40\mp 0.05$	3723 ± 30	199 pm (106 GHz)	1.07	1.45701 ± 0.00006
4	636 ± 5	2.2	1.15 × 10^7^	$398.11\mp 0.05$	3857 ± 30	192 pm (102 GHz)	2.94	1.45465 ± 0.00004
5	554 ± 5	1.4	1.18 × 10^7^	$400.44\mp 0.05$	3359 ± 30	220 pm (117 GHz)	3.45	1.44589 ± 0.00004
6	345 ± 5	1.1	5.19 × 10^7^	$403.23\mp 0.07$	2092 ± 30	354 pm (189 GHz)	24.5	1.43589 ± 0.00005
7	713 ± 5	0.3	8.73 × 10^6^	$427.92\mp 0.03$	4324 ± 30	171 pm (91 GHz)	1.99	1.35332 ± 0.00001
8	404 ± 5	1.0	1.46 × 10^6^	$404.29\mp 0.06$	2450 ± 30	302 pm (161 GHz)	0.59	1.43241 ± 0.00003
9	231 ± 5	NA	1.54 × 10^6^	NA	NA	528 pm (282 GHz)	1.09	NA

**Figure 4:**
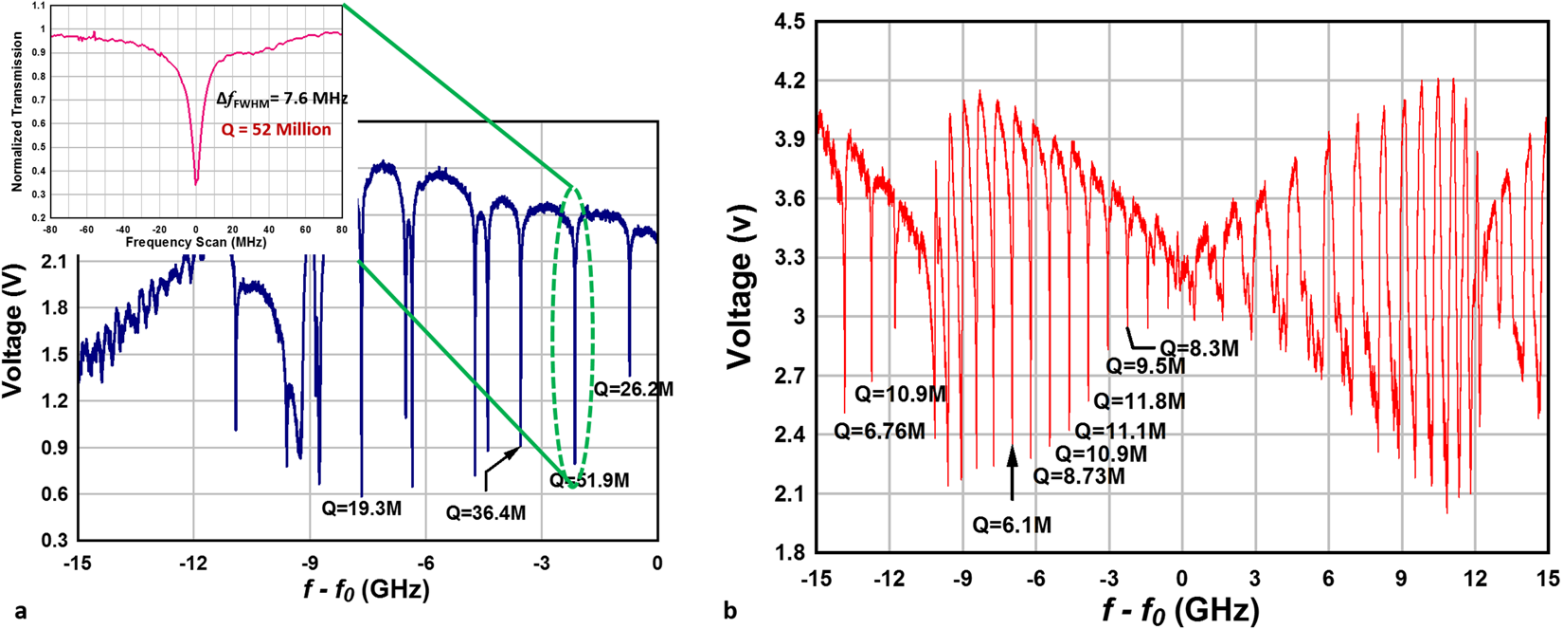
Transmission spectrum of the optical resonance in (**a**) microspherical shell #6 and (**b**) microspherical shell #5 within 15 GHz frequency span.

The small mode volume of microspherical shell #6, diameter of 345 μm and thin sidewall thickness of 1.1 μm, results in less than 10 observed resonance modes in the transmission spectrum within the 15 GHz frequency span as shown in Fig. 4(a). Asymmetry in the transmission spectrum was observed upon scanning the laser frequency up and down as shown in Fig. 4(b) and arises from thermally induced linewidth broadening/compression effect in optical micro-resonators^34,35^. The inset image of Fig. 4(a) shows a resonance mode with a very high *Q-*factor of 5.19 × 10^7^ which was deduced by fitting a Lorentzian curve to the transmission spectrum. For this resonance mode, the calculated finesse was 2.45 × 10^4^. For the microspherical shell resonator #5, the resonance spectrum shows equally-spaced resonance frequencies in the transmission spectrum of as shown in Fig. 4(b). Since the free spectral range for this microbubble was 117 GHz, these peaks with a frequency spacing of 0.76 GHz must arise due to azimuthal mode splitting. Azimuthal mode splitting typically arises from the removal of degeneracy of polar quantum number *l* in the solution of the spherical harmonic mode function^36^ due to eccentricity of the microbubbles. The analytical expression for azimuthal mode splitting is derived using perturbation method and is given by^37^

7 $$\frac{{\rm{\Delta }}{f}_{ecc}}{{f}_{nml}}=-\frac{\varepsilon }{6}(1-3\frac{{|m|}^{2}}{l(l+1)})$$

where *ε* is the eccentricity of the microspherical shell, *f*_*nml*_ is the resonance frequency of the mode with radial mode number *n*, azimuthal mode number *m*, and polar mode number *l*. For a shell with polar radius *r*_*p*_ and equatorial radius *r*_*e*_, eccentricity *ɛ* is defined as $\varepsilon =\frac{{r}_{p}-{r}_{e}}{{r}_{e}}$. Hence, the splitting between successive azimuthal mode numbers within the free spectral range can be given by^38^

8 $${\rm{\Delta }}{f}_{ecc}\equiv |\,{f}_{nml}-{f}_{n,m+1,l}|\approx {f}_{nml}\cdot \varepsilon \frac{|m|+1/2}{{l}^{2}}$$

The resonance frequency of microspherical shell #5 was calculated by COMSOL^®^ simulation to be *f*_*nml*_ = 400.437508 THz under the assumption of an ideal spherical shell of uniform wall thickness and a radius of 277 μm. Using the equation for resonance condition, for microbubble #5, and λ = 760 nm, the azimuthal mode number can be calculated to be *m* = 3359. Under the assumption, that the observed peaks in Fig. 4(b) are due to the splitting of the fundamental TE azimuthal mode (*m* ≈ *l* ≈ *3359*), the frequency spacing of 0.76 GHz leads to a corresponding eccentricity of ɛ ≈ 0.65%. Dimensional data of microspherical shell #5 from Table 1, can be used to calculate value of eccentricity which gives a value of 2.5%. This is ~4 times larger than the eccentricity estimated using eq. (8). The large uncertainty of ~5 μm in determining the microspherical shell diameter and height using optical images can easily account for the observed discrepancy and therefore, the two eccentricities can be considered to agree well within the errors of the measurements.

### Temperature Sensitivity of Optical Resonance in Microspherical Shells

From the WGM resonance condition, it is clear that the resonance frequencies depend on both the size and refractive index of the resonator. A small change in the size or the refractive index can cause a significant resonance frequency shift. Since both the refractive index and the size of the microspherical shells depend upon temperature due to thermo-optic and thermal expansion effects, a WGM resonator can be configured as a sensitive thermometer. Assuming a linear dependence of thermal expansion and refractive index for small temperature variations, these can be expressed as d*r*/*r* = *αdT* and *dn*_*r*_ = *βdT*; where *α* and *β* are the temperature coefficient of expansion (TCE) and thermo-optic coefficient respectively of borosilicate glass. Taking a variation of the resonance condition, we can now express the fractional change in the frequency as

9 $$\frac{d{f}_{nml}}{{f}_{nml}}=-(\frac{d{n}_{r}}{{n}_{r}}+\frac{dr}{r})=-(\frac{\beta }{{n}_{r}}+\alpha )dT\Rightarrow \frac{d{f}_{nml}}{{f}_{nml}dT}=-(\frac{\beta }{{n}_{r}}+\alpha )$$

**Figure 5:**
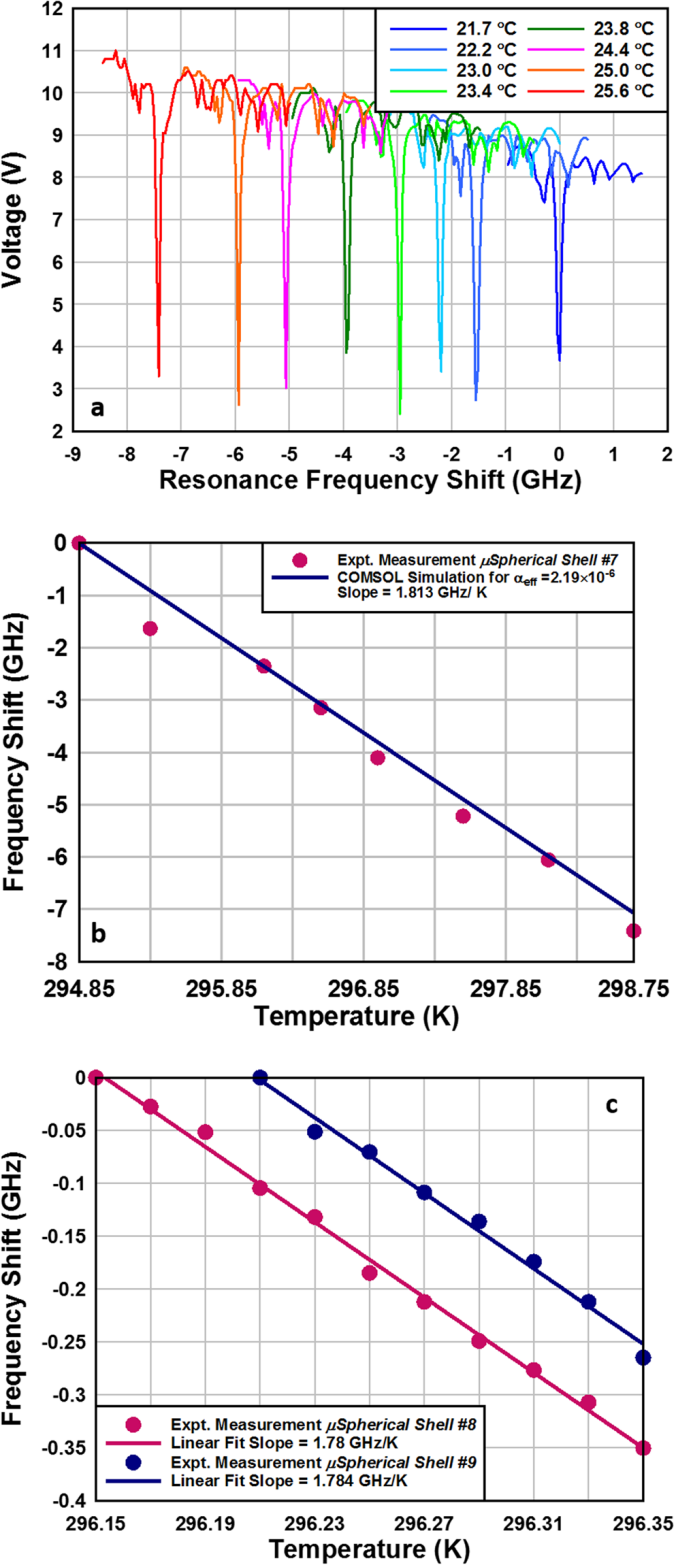
(**a**) Experimentally measured temperature induced resonance frequency shift of ~10^7^ *Q-*factor resonance mode in the transmission spectrum of microspherical shell #7. (**b**) COMSOL simulation was used to fit the experimentally measured frequency shift by parametrically tuning the effective value of TCE of the microspherical shell. Good fit was found for an effective TCE value of 2.19 × 10^−6^ K^−1^ for the microspherical shell #7. (**c**) Measured temperature induced resonance frequency shift within a finer temperature change for microspherical shell #8 and #9. These two resonators are located on the same chip and were fabricated and blown under identical conditions.

The frequency shift per unit change in the temperature of the microspherical shell can be estimated using eq. (9) by using borosilicate material properties at λ = 760 nm, i.e., thermo-optic coefficient *β* = 3.41 × 10^−6^ K^−1^ ^39,40^, temperature coefficient of expansion *α* = 3.25 × 10^−6^ K^−1^ ^41^, and *n*_*r*_ = 1.467^42^. This gives a theoretical frequency shift of 5.574 ppm K^−1^. The sensitivity of the microspherical shells to temperature changes was experimentally measured by placing the device on the hot side of a calibrated Peltier cooler. WGM mode of microspherical shell #7, with a *Q*-factor of ~10^7^, was monitored as a function of temperature. As seen in Fig. 5(a), the resonance frequency decreases with increasing temperature. The induced frequency shift as a function of temperature, Fig. 5(b), shows a linear dependence with an outstanding thermal sensitivity of −1.81 GHz K^−1^ (equal to a wavelength shift of −3.48 pm K^−1^) and corresponds to fractional frequency temperature sensitivity of −4.23 × 10^−6^ K^−1^. Assuming the frequency resolution of measurement system to be 100 kHz at a *Q-*factor of 10^7^, the microspherical shell temperature resolution can be determined to be 55 µK. For microspherical shell #7, the resonance frequency *f*_*nml*_ was first calculated using COMSOL^®^ modeling at 20 °C. Thereafter, using the temperature coefficient of expansion and the thermo-optic coefficient, the microspherical shell dimensions and refractive index were changed to the corresponding values at the increased temperature and the new *f*_*nml*_ was modeled. Through this method, the expected frequency change was modeled through the range of the experimental temperature values and resulted in a modeled slope of −2.23 GHz K^−1^. Clearly the ideal model overestimates the slope in comparison to the obtained experimental slope of −1.81 GHz K^−1^. The values measured in this work agree very well to those reported on solid silica microspheres where the authors reported a temperature sensitivity of frequency of 1.808 GHz/K at a wavelength of 1530.335 nm for a 430 μm bead^43^. It must be noted that the ultimate change in the microspherical shell equatorial radius is not only a function of the TCE of the glass bubble but is also affected by the TCE mismatch between the borosilicate glass and the bonded silicon substrate at the base. To account for these issues, we parametrized the effective TCE of glass and modeled the frequency shift to match the experimental data. As shown in Fig. 5(b), a near ideal fit was obtained by using an effective TCE of borosilicate glass, *α*|_eff_ = 2.19 × 10^−6^ K^−1^. Figure 5(c) shows the measured thermal sensitivity of microspherical shells #8 and #9 performed with a much finer temperature scan. The experimentally obtained linear slopes for these silica shells of 1.78 GHz K^−1^ is very similar to that obtained for microspherical shell #7. It must also be noted that the microspherical shells #8 and #9 are located on the same chip and, although of significantly different dimensions, show near identical thermal dependence of resonance frequency shift. This can be considered as further evidence of the fact that the effective TCE of the microspherical shells sensitively depends upon the stresses induced in the between the glass and silicon substrates during bonding as well as the temperature at which the glass shells are blown.

### Dependence of Optical Resonance in Microspherical Shells on Refractive Index Changes

**Figure 6:**
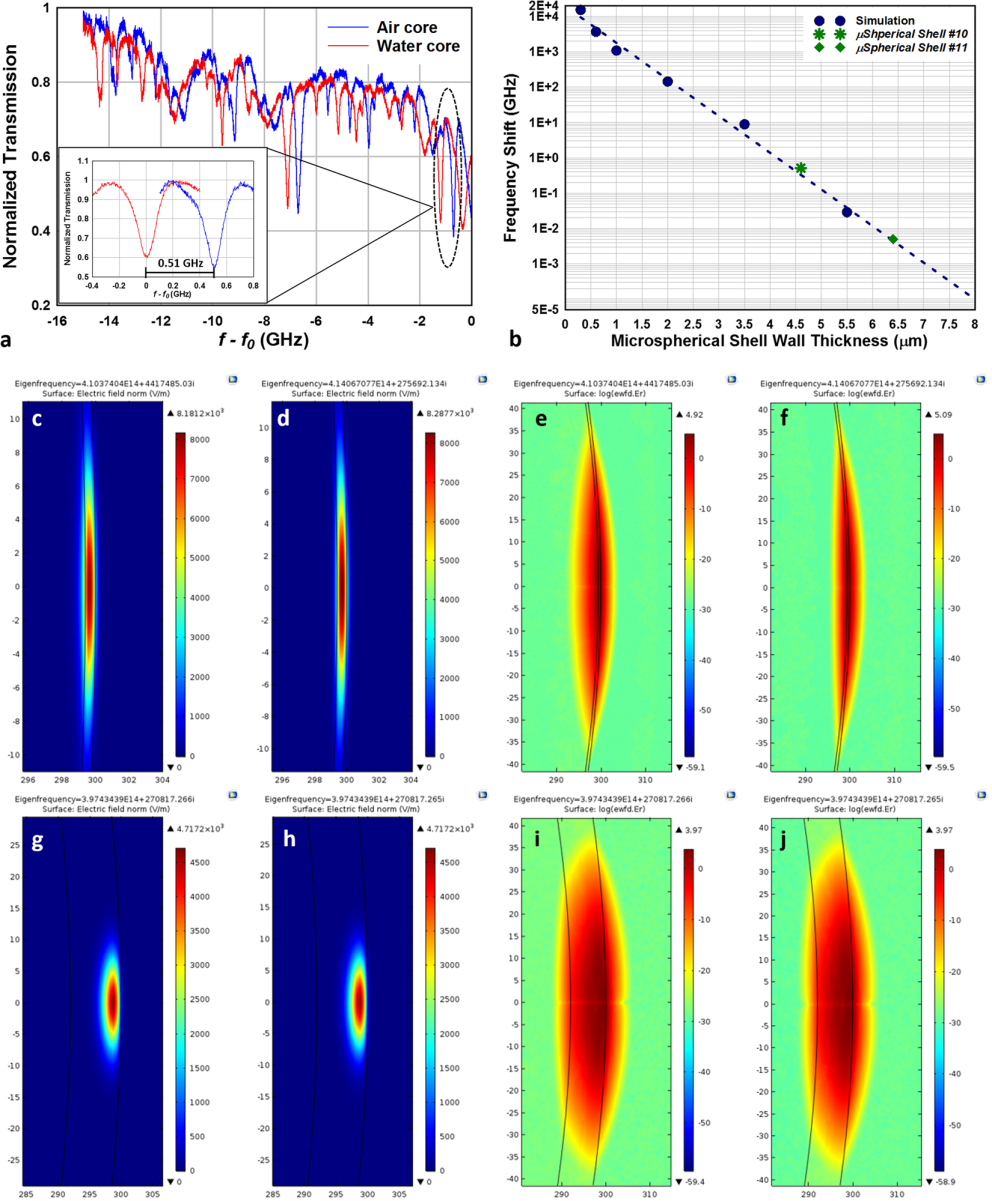
(**a**) Transmission spectrum of resonant modes obtained from *microspherical shell #10* with wall thickness of 4.7 µm. A blue-shift of the resonant modes was observed as the water-filled microspherical shell core dries out. Inset image shows 0.51 GHz frequency shift observed in a 2.5 × 10^6^ *Q-*factor mode. (**b**) COMSOL simulated frequency shifts between water-core and air-core microspherical shells with diameters of 600 µm as a function of the shell thicknesses ranging from 300 nm to 10 µm. Experimental data for two microspherical shells of thicknesses 4.7 μm and 6.4 μm is also shown. (**c**,**d**) FEM solved fundamental TE mode showing the spatial distribution of the electric field intensity in 0.6 µm shell thickness with water and air core respectively. (**e**,**f**) Electric field intensity is plotted in logarithmic scale for water and air cores in 0.6 µm thick shell and clearly exhibits penetration of electric field into water core in (**c**). (**g**,**h**) FEM solved fundamental TE mode in a 8 µm thick microspherical shell with water and air core respectively. (**i**,**j**) Electric field intensity plotted in logarithmic scale for the two cores for the 8 µm thick microspherical shell. The simulations clearly show that the TE mode electric field interacts strongly with the fluid in the core of thinner walled microspherical shells than for thicker shell walls and explains the larger frequency shift obtained for thinner walled shells.

A major advantage of WGM resonators consisting of hollow shell structures is that fluidic analyte samples can be introduced and made to interact with the optical resonance mode through the inner surface of these structures^8^. As we have already demonstrated, through microfabrication processes the thickness and the diameters of the microspherical shells can be precisely controlled and reproduced. Sensitivity to the fluid contained in the inner volume of the optofluidic microspherical resonator as a function of the shell wall thickness was experimentally examined. For these experiments, on-chip glass microspherical shells were coated and protected with crystal bond epoxy on the outer surface and the silicon substrate was etched and thinned in potassium hydroxide solution to a thickness of 250 µm and until a backside access hole to the inner surface of the microspherical shell was obtained. The crystal bond protective coating was thereafter removed by dissolving it in acetone at 80 °C. With open access to the shell cavity, the microspherical shell was filled with water (Refractive index *n*_*water*_ = 1.332986 at 20 °C and 760 nm) in a vacuum chamber. The filled water within the microspherical shell was held inside the cavity in atmosphere due to surface tension at the small opening. However, the water gradually evaporated and eventually dried out in the microspherical cavity. When the water in the micro-bubble evaporates away, the resonance frequency blue shifts due to a decrease of the effective refractive index due to the interaction of optical resonance on the inner surface. The water-filled microspherical shells #10 a with a wall thickness of 4.7 µm and #11 with a wall thickness of 6.4 µm were coupled with fiber taper and the resonance modes were monitored and tracked as the water dried out in the microspherical shells in real-time. The transmission spectrum of microspherical shell #10, in Fig. 6(a), shows a blue-shift due to the decrease in the effective refractive index inside the microbubble cavity as a consequence of the water drying out and being replaced by air. Inset in Fig. 6(a) shows a zoomed-in image of a shifted resonance mode with and without water in the microspherical shell. A frequency shift of 0.51 GHz and an increase in the *Q-*factor from 2.51 × 10^6^ to 2.69 × 10^6^ was observed as the core changed from water to air. Resonance frequency shift was barely observed in the transmission spectrum of microspherical shell #11. The shift in the resonance frequency of the first radial order fundamental TE mode between water core and air core of a 600 µm diameter microspherical shell resonator was simulated as a function of the shell wall thicknesses using COMSOL^®^ and is shown in Fig. 6(b). Experimentally measured frequency shifts of microspherical shells #10 and #11 are in good agreement with the COMSOL^®^ simulations. The very small frequency shift observed for microspherical shell #11 is due to the much larger shell wall thickness of 6.4 µm. The electric field confinement in a 0.6 µm thick shell at the fundamental eigenfrequency is shown in Fig. 6(c) for water filled and in Fig. 6(d) for air filled shell cores. Figure 6(e) and (f) plot the intensity of the electric field on log scale for the 0.6 µm thick shell and show that the electric field clearly penetrates into the water core within the microspherical shell. On the other hand, Fi. 6(g) and (h) show that the electric field is entirely confined to inside the 8 µm thick glass shell with minimal interaction with the fluid contained in the cavity of the microspherical shell. Figure 6(i) and (j) show the electric field intensity for the 8 µm shell on log scale. Thus, thicker walled shells are expected to show little sensitivity to any fluidic core changes or interactions.

## Conclusions

In this work, we have demonstrated optical resonance in microspherical glass shells fabricated on a silicon substrate using microfabrication methods. The chip-scale glass blowing technique allows for the fabrication of microspherical shells with radii ranging from 0.2 mm–1 mm and thicknesses ranging from 0.3 μm–10 μm. The paper explains the effect of the various processing parameters on the final dimensions of the glass microspherical shells. The fabrication method described is highly reproducible for manufacturing the resonators with very high dimensional control and tolerance. The microspherical glass shells have been shown to be excellent optical resonators with *Q-*factors in excess of 50 million. These optical resonators can be used as ultrahigh sensitivity temperature sensors with possible resolutions of 55 μK. The small thermal mass and integration with on-chip microfluidic channels can allow these to be configured into sensitive lab-on-a-chip devices. The modulation of the effective refractive index in thin walled (≤1 1 μm) microspherical shells through the introduction of fluids with varying refractive index in the inner core or by selective adsorption of various molecules on the inner walls, is likely to provide a very sensitive and reproducible platform for bioanalyte detection and lab-on-a-chip applications. The microspherical shells are also sensitive to forces on the bubble structure and based on this principle sensitive magnetometers have been recently investigated^44^. In summary, the presented chip-scale, borosilicate glass, microspherical shell resonators provide a very sensitive platform for various applications including temperature, vapor pressure, motion, pressure, bioanalytes, and optomechanical sensing.
